# American Academy of Dental Science

**Published:** 1882-01

**Authors:** 


### AMERICAN ACADEMY OF DENTAL SCIENCE.

The Fourteenth Annual Meeting of the American Academy of Dental Science was held October 26, at the Hotel Vendome, Boston, Mass., the President, J. L. Williams, M. D., in the chair. President Williams, on calling the meeting to order, said:

"This society has only arrived at its fourteenth birthday, but it is old enough to profit by experience. It has seen simple manual skill grow to become less rare than it was two decades ago; though its esthetic application is yet far too uncommon. And there is reason for congratulation on the present awakening of the conviction, held by the founders of dental surgery, that sound opinions and safe practice may be expected only from a thorough knowledge of the principles and sympathies that in health and disease govern the human system, of which the mouth is a most important part. And, I may add, without this knowledge, the finest manual skill may be useless, or, indeed, mischievous. For the axiom in engineering is equally true in this practice, that the greater the skill misapplied the more aggravating the blunder. But by a general zeal for knowledge of the fundamental principles of practice, we may hope for a higher advance than ever. And it is mainly by such knowledge that this academy can deserve its name as a scientific body."

After the reading of the minutes of the previous meeting the reports of the secretary and treasurer was presented, the former showing that there are now on the roll the names of thirty-two members:

Appropriate resolutions on the death of Dr. Daniel Harwood, one of the oldest members, were adopted.

The following named officers were elected:

*President.*—Dr. T. H. Chandler, Boston.

*Vice-President.*—Dr. George T. Moffatt, Boston.

*Recording Secretary.*—Dr. H. F. Hamilton, Boston.

*Corresponding Secretary.*—Dr. E. B. Hitchcock, Boston.

*Treasurer.*—Dr. L. D. Shepard, Boston.

*Librarian.*—Dr. H. C. Merriam, Salem, Mass.

*Censors.*—C. P. Wilson, J. H. Batchelder, and J. T. Codman.

The annual address was delivered by Dr. F. N. Seabury, of Providence, R. I. At the close of the exercises the annual dinner was served.—*Dental Cosmos.*

